# The steroid benefit in treating complicated haemangioma

**DOI:** 10.4103/0970-0358.59290

**Published:** 2009

**Authors:** Kamal H. Saleh

**Affiliations:** Department of Plastic Surgery, Al Emadi Hospital, Doha, Qatar

**Keywords:** Steroid therapy, haemangiomas, diluted steroid

## Abstract

The clinical study included 30 patients with complicated cutaneous haemangioma (ulceration, bleeding, obstruction of anatomical orifices, and interference with function or movement). The patients were studied regarding the age group, sex, site of lesion, size of lesion, and the percentage of regression after treatment with steroid.

The age ranged from three months to six years, there were 20 female patients and 10 male patients. We used local injection of diluted triamcinolone 4 mg with 5 ml. 0.9% NACI (normal saline), injected through 23-guage syringe under local or general anaesthesia every two weeks for six to eight sessions depending on the severity of the case, followed by a local pressure dressing. We measured the size of the lesion before each session and recorded the regression of the lesion. The patients were followed up for two years. Haemangioma commonly presents in infants and children, most commonly in females, especially in the head and neck and are usually of a small size. It regresses if the treatment is started earlier.

## INTRODUCTION

Haemangiomas are immature rests of vasoformative tissue that demonstrate angioblastic proliferation and regression and represent the most common vascular tumour of childhood.[1] Infantile haemangioma is more common in females, occurs in 10% of the children, usually appears within a few weeks after birth; about 30-90% of cases undergo a characteristic proliferation phase that lasts between 6–12 months. This proliferative period is followed by a stable phase, and finally a period of regression or involution, occurs[2] usually between the 10-12 years.[3] Infants with alarming cutaneous haemangioma may be treated medically with a high dose of steroid. However, only two-thirds of these haemangiomas regress or stabilize[4] and well defined surgery is may be necessary for aesthetic correction at the age of 8-10 years[5] or to improve sign and symptoms of infantile hemangiomas.[6] The size of the haemangioma and the age of initiation of the treatment are the most important factors affecting the response to the treatment.[7] The site of the lesion and the phase of the haemangioma are also factors that affect outcome.[8] The age of initiation to steroid is usually at 7.5 months and the treatment may continue for as long as five months.[9] Selection of the type of steroid and the route of administration and dose schedule is guided by clinical experience.[10] Intralesional injection of steroid is an effective treatment for haemangioma of the head and neck,[7] with injection pressure routinely exceeding the systemic arterial pressure in capillary haemangiomas.[11]

## MATERIAL AND METHODS

Thirty patients with complicated haemangioma (ulceration, bleeding, obstruction of anatomical orifice, and interference with function or movement) of different age groups collected through simple random sampling in outpatient cases were treated with local injection of diluted triamcinolone 4 mg with 5 ml normal saline in multiple sessions, two weeks apart [Figure 1a,b,c and Figure 2a,b]. The age of patients ranged from three months to six years; 20 patients were females while 10 were males. We used traditional syringe for injection (23-guage) under local or general anaesthesia, followed by local pressure dressing and repeated the procedure every two weeks for six to eight sessions depending on the severity of case,. The size of the lesion before each session was measured to record the regression of the lesion.

**Figure 1a:**
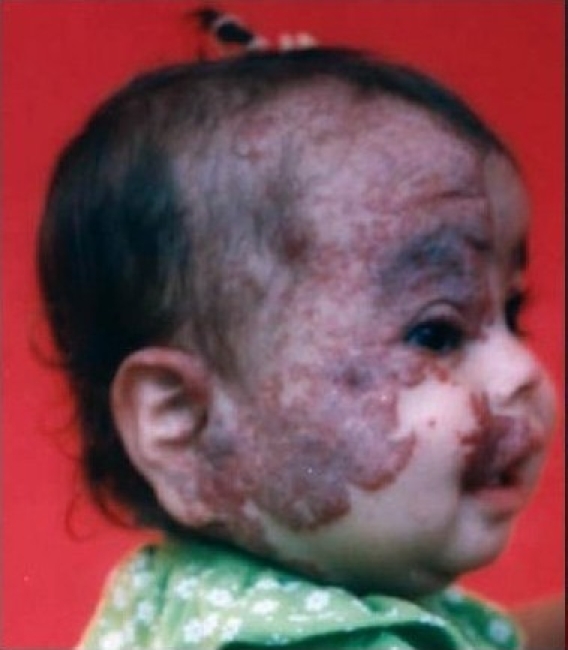
First patient before injections

**Figure 1b:**
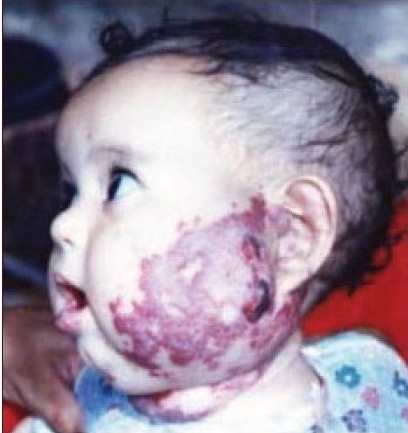
Second patient before injection of steroid

**Figure 1c:**
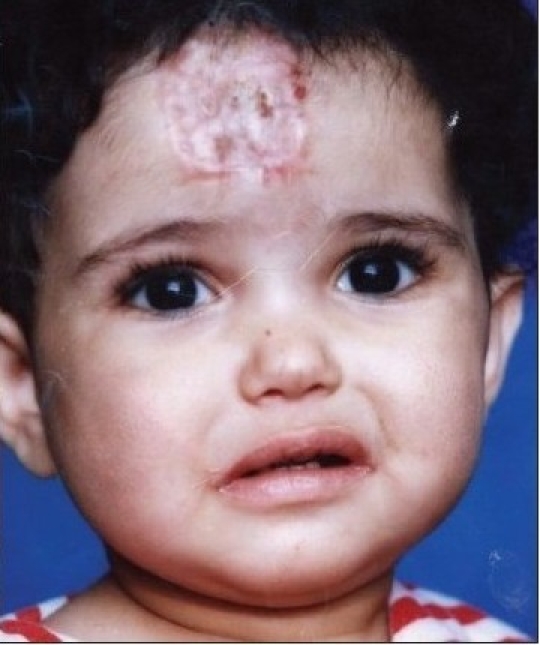
After 12 months from the injection

**Figure 2a:**
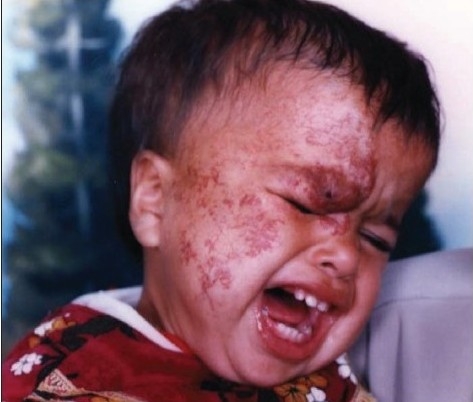
After injection of steroids

**Figure 2b:**
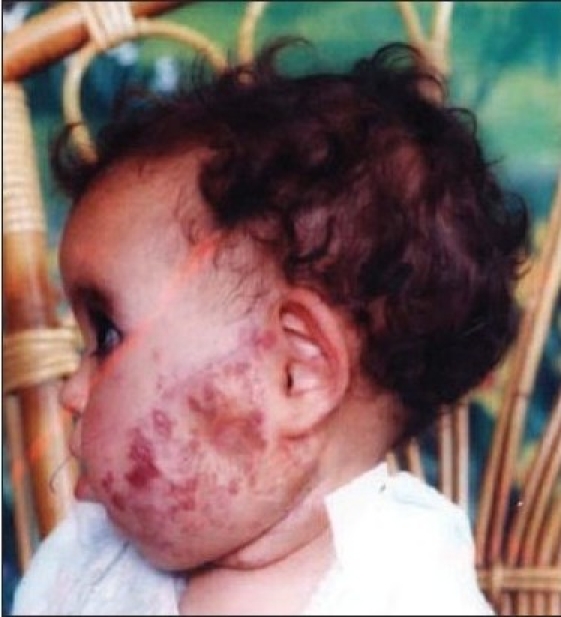
Patient after injection of steroids

## RESULTS

Of the 30 patients that visited the outpatient clinic, 20 were females while 10 patients were males.

The age of presentation to the clinic was: 10 patients < one year, 10 patients from one year - < four years, and 10 patients from four years - < six years.

The most common site of haemangioma in these patients was head and neck in about 51%, the second most common site was the trunk (33%), followed by the extremities (13%), and the genitalia (3%).

We found also that 10 patients the size of cutaneous hemangioma was (1-2cm), 8 patients was (2-3cm), 7 patients was (3-4cm),& 5 patients was (4-5cm), as shown in the Table 1.

**Table 1:** Size of the cutaneous hemangioma

*Number of cases*	*Size in cm*
10 cases	1-2cm
8 cases	2-3 cm
7 cases	3-4 cm
5 cases	4-5 cm

After treatment we found that the 60% of the cutaneous haemangiomas regressed in the age group between one-<two years old, in the two to four years age group, they regressed in about 40%, and in >six years old, the regression was seen in about 25% as shown in Table 2.

**Table 2:** The percentage of regression according to the age group and number of cases

*Age of patient*	*% of regression*	*No. of patients*
1 - <2 years	60	10
2 – 4 years	40	10
>6 years	25	10

We found that only three patients suffered from complication of steroid treatment, moon facies in two and systemic infection in one.

## DISCUSSION

In 30 patients with cutaneous haemangioma, we found that the most common age group presenting with haemangiomas was that of infants and children (three months - six years). This deduction agrees with the study of Winter *et al*., who found that 65.3% were infants and children.[3]

The female to male ratio was 2:1, and is similar to the study of Garzon M who found that haemangiomas occur in females three times more than males.[9]

Haemangiomas presented in the head and neck region in about 51%, trunk (33%), extremities (13%), and genitalia (3%). This agrees with the study conducted by Mullkin and Glowacki, who found that 60% of haemangiomas are in the head and neck area, 25% in the trunk, and 15% in the extremities.[12]

Like the Garzon study[9] we also found that a majority of our haemangiomas, 83.3%, were smaller in size.

After treatment with local steroid the percentage of regression was found to be 60% when we started the treatment in the early period (three months - <two years), 40% from two - <four years and 25% from four – six years. This is similar to the study of Akyus *et al*., who found that the age of initiation of treatment is the most important factor affecting the response to treatment.[1]
